# Special Issue on Molecular and Translational Research on Colorectal Cancer 2.0

**DOI:** 10.3390/ijms22147479

**Published:** 2021-07-13

**Authors:** Alessandro Passardi, Emanuela Scarpi, Paola Ulivi

**Affiliations:** 1 Department of Medical Oncology, IRCCS Istituto Romagnolo per lo Studio dei Tumori (IRST) “Dino Amadori”, Via P. Maroncelli 40, 47014 Meldola, Italy; 2 Unit of Biostatistics and Clinical Trials, IRCCS Istituto Romagnolo per lo Studio dei Tumori (IRST) “Dino Amadori”, Via P. Maroncelli 40, 47014 Meldola, Italy; emanuela.scarpi@irst.emr.it; 3 Biosciences Laboratory, IRCCS Istituto Romagnolo per lo Studio dei Tumori (IRST) “Dino Amadori”, Via P. Maroncelli 40, 47014 Meldola, Italy; paola.ulivi@irst.emr.it

**Keywords:** colorectal cancer, biomarkers, heterogeneity, liquid biopsy, immunotherapy

## Abstract

The present editorial aims to summarise the six scientific papers that have contributed to this Special Issue, focusing on different aspects of molecular and translational research on colorectal cancer. We believe that the present Special Issue might contribute to the expansion of the current knowledge concerning potential molecular predictive and/or prognostic biomarkers in CRC, as well as new targets for anticancer treatment. This may help in identifying new strategies to improve diagnostic and therapeutic approaches.

Colorectal cancer (CRC) is the fourth leading cause of cancer mortality worldwide. As other cancers, it is a genetic disease, determined and fuelled by the succession of somatic mutations over the course of life by normal cells and capable of altering processes essential for life and cell death. Therefore, a deep analysis of the several signalling pathways involved in CRC is needed to elucidate the underlying mechanism of CRC progression and metastasisation [1]. The review presented by Ahmed Malki et al. [2] focused on several cellular signalling pathways (Wnt/β-catenin, p53, TGF-β/SMAD, NF-κB, Notch, VEGF, and JAKs/STAT3), that have been associated with CRC progression and metastasis, as well as the alterations in methylation patterns. Only a clear understanding of the molecular mechanisms that lead to CRC growth can pave the way for the development of novel diagnostic and therapeutic strategies.

Among tumour-related mutations, aberrant activation of WNT/β-catenin signalling is very common and indispensable for CRC initiation and progression. For this reason, it appears as a promising target for developing novel CRC therapeutics. In this field, several derivatives of Hispolon (a fungal-derived polyphenol with anticancer effect), including dehydroxyhispolon methyl ether (DHME), have been chemically synthesised. Hueng-Chuen Fan et al. [3] investigated the antitumor effects of DHME in CRC cells. DHME provoked CRC cell apoptosis via suppression of the WNT/β-catenin signalling axis and appeared to be a selective proapoptotic agent with more potent cytotoxicity than Hispolon.

Holliday junction recognition protein (HJURP), a centromeric protein that functions as a specific chaperone for centromere protein-A (CENP-A), might have a role in cancer initiation and progression. Dong Hyun Kang et al. [4] conducted a pathologic evaluation of HJURP expression in surgically resected CRC using immunohistochemistry (IHC), followed by a functional evaluation of HJURP knockdown by siRNA in four CRC cell lines (HT29, HCT116, SW480, SW620). A higher overall survival rate was associated with a high IHC expression of HJURP; moreover, the knockdown of HJURP suppressed the proliferation, migration, invasion, and tumorigenicity of CRC cells. These results indicated HJURP as a new potential prognostic biomarker and a target for drug discovery.

Similarly, Fee Klupp et al. [5] analysed expressional differences of granulin in CRC tumoral tissue and healthy colon mucosa using qRT-PCR and IHC. Granulin was over expressed in tumoral tissue compared to healthy tissue; moreover, the overexpression of granulin was associated with a significantly decreased overall survival, and in vitro downregulation of granulin by siRNA in HCT-116 cells significantly diminished their invasive capacities. The authors concluded that granulin might represent a prognostic biomarker and a potential target for a tailored anticancer treatment.

Immunotherapy represents a major area of CRC treatment development. However, in too many patients, the underlying mechanisms of immunotherapy resistance remains elusive. Yulia I. Nussbaum et al. [6] provided an interesting review of recent studies analysing anti-tumour immunity in CRC, and examined new immune contexture assessment methods that might reveal new information important for immune therapy insights. Next-to-next-generation sequencing and big data technologies, single-cell transcriptomic, genomic, and proteomic analysis, such as scRNA-seq, as well as spatial transcriptomics might be useful in expanding our ability to evaluate the immune landscape of cancers.

In summary, the field of molecular and translational research on CRC is growing rapidly with an important contribution to the knowledge on the mechanisms of carcinogenesis, metastasisation and response or resistance to anticancer treatments. The papers submitted to this special issue could stimulate the deepening of research on new tumour targets in CRC and help identifying new strategies to improve diagnostic and therapeutic approaches.

## Data Availability

Not applicable.

## References

[1] Siskova A., Cervena K., Kral J., Hucl T., Vodicka P., Vymetalkova V. (2020). Colorectal Adenomas—Genetics and Searching for New Molecular Screening Biomarkers. Int. J. Mol. Sci..

[2] Malki A., ElRuz R., Gupta I., Allouch A., Vranic S., Al Moustafa A. (2021). Molecular Mechanisms of Colon Cancer Progression and Metastasis: Recent Insights and Advancements. Int. J. Mol. Sci..

[3] Fan H., Hsieh Y., Li L., Chang C., Janoušková K., Ramani M., Subbaraju G., Cheng K., Chang C. (2020). Dehydroxyhispolon Methyl Ether, A Hispolon Derivative, Inhibits WNT/β-Catenin Signaling to Elicit Human Colorectal Carcinoma Cell Apoptosis. Int. J. Mol. Sci..

[4] Kang D., Woo J., Kim H., Kim S., Ji S., Jaygal G., Ahn T., Kim H., Kwak H., Kim C. (2020). Prognostic Relevance of HJURP Expression in Patients with Surgically Resected Colorectal Cancer. Int. J. Mol. Sci..

[5] Klupp F., Kahlert C., Franz C., Halama N., Schleussner N., Wirsik N., Warth A., Schmidt T., Ulrich A. (2021). Granulin: An Invasive and Survival-Determining Marker in Colorectal Cancer Patients. Int. J. Mol. Sci..

[6] Nussbaum Y., Manjunath Y., Suvilesh K., Warren W., Shyu C., Kaifi J., Ciorba M., Mitchem J. (2021). Current and Prospective Methods for Assessing Anti-Tumor Immunity in Colorectal Cancer. Int. J. Mol. Sci..

